# Access to an Electronic Health Record: A Polish National Survey

**DOI:** 10.3390/ijerph17176165

**Published:** 2020-08-25

**Authors:** Maria Magdalena Bujnowska-Fedak, Łukasz Wysoczański

**Affiliations:** Department of Family Medicine, Wroclaw Medical University, 51-141 Wrocław, Poland; lukasz.wysoczanski@student.umed.wroc.pl

**Keywords:** electronic health record (EHR), e-health, telemedicine, year-on-year trends, national survey

## Abstract

In Poland, as in many countries around the world, e-health services are becoming more and more popular. Obligatory e-sick leave was implemented, followed by e-prescriptions and e-referrals. Therefore, it is worth considering the introduction of a complete electronic health record (EHR) that can be accessed by doctors and patients. The main aim of the study is to find out whether patients want to have access to their EHRs and if they would agree to pay for such a service. The research was based on three surveys conducted among 1000 Polish adults in 2007, 2012, and 2018. The sample collection was carried out by the national opinion poll agency, with the use of computer-assisted telephone interviews. Over 60% of respondents were interested in the possibility of accessing EHRs in general, and almost 50% of them were ready to pay for it. Nevertheless, when analyzing all the year-on-year trends, they were subject to a gradual decrease. The youngest age group was the one most interested in EHRs, while the group comprising respondents in middle age was the one most willing to pay for it. There is still great potential in implementing EHRs on a bigger scale.

## 1. Introduction

Currently, we are undergoing a digital revolution that dominates most areas of our life. We are experiencing this more distinctly in medicine. The vast majority of the population possess telephones, tablets, and computers with internet access. These devices are used, among other functions, for health-related purposes [1]. E-health services are becoming more and more a necessity [1]. Especially in the current situation, with the pandemic of SARS-CoV-2 announced by the World Health Organization (WHO), e-health services play a key role in the functioning of primary and outpatient healthcare [2]. The trend associated with the increasing use of e-health services has been observed all over the world. For example, e-prescriptions have been available in the Netherlands since 1998 [3], in Great Britain since 2003 [4], in the USA since 2008 [5,6], and in the Czech Republic since 2020 [7]. In Poland, obligatory e-sick leave was implemented in 2019, and then e-prescription was introduced. Since 2020, it has been the only form of prescription used in everyday medical practice [8,9]. The introduction of e-referrals is planned for 2021, and the system is currently operating on a pilot platform [9]. In the beginning, these solutions aroused some fears and aversion, but ultimately they have greatly improved the Polish healthcare system and their function has been permanently adopted [8]. All these e-health services comprise a complete electronic health record (EHR), which provides uniform access to medical documentation for healthcare specialists and patients, gathering as much data as possible in one system. According to WHO, national EHRs work successfully in many countries, for example, the Netherlands, the United Kingdom, Italy, Germany, Scandinavia, Canada, and Australia [10,11], as well as in the United States [12]. Despite the much earlier introduction of electronic health services in some countries, the usage of EHRs is still at a development stage and is constantly being improved [3,12,13,14]. However, the implementation and use of EHRs are certainly challenging. Its deployment is associated with the learning of new IT systems, ensuring the computerization of all medical facilities and security of data collection and transmission [15]. The United States was one of the first countries to introduce EHRs, which constitutes one of the most developed and refined medical databases. There has been a significant upward trend in the use of this system over the years [16]. In Europe, EHRs are being developed in many countries. The function and assumptions of these systems are slightly different, but the European Union is trying to introduce general standards for them within the European community [14]. Standardization and widespread distribution of EHR systems improve the work of medical personnel in the interdisciplinary care of patients, enable better medical and insurance-allowance supervision, increase patient safety, and serve as financially beneficial solutions for the healthcare systems [17]. In Poland, the requirement that EHRs should be mandatory is gradually being introduced and increasingly extended. Having that in mind, the aim of this research project is to find out whether Polish patients want to have access to their EHRs and to determine if they would agree to pay for such a service. Analysis of the trends concerning interest in EHRs is also carried out.

## 2. Materials and Methods

### 2.1. Study Design

The research project was based on three national surveys conducted in the years 2007, 2012, and 2017/2018. Each survey was carried out by a national opinion poll agency (Kantar TNS and TNS-OBOP) among 1000 Polish adults using computer-assisted telephone interviews (CATIs). The total number of respondents in the three analyzed surveys was 3000 people. The course of the study was constantly monitored by experienced supervisors who controlled the work of the interviewers in a CATI studio. While selecting telephone numbers, an equal distribution was maintained in terms of age, sex, size of the place of residence, and the region of the country. Both landlines and cell phones were included. During the study, the geographical distribution for the voivodships and the size of the cities/villages for the entire sample was controlled in order to ensure its representativeness. The respondents were selected on the basis of compliance with the imposed quotas, ensuring that the respondents with the required sociodemographic characteristics were reached. The sample was selected in terms of parameters as a nationwide, representative sample of adults, then stratified according to the voivodship and size of the place of residence, and quoted according to sex and age. A nonresponding group included incorrect numbers or cases of not answering the phone, people who did not want to participate in the interview, and those who were too sick to take part. The vast majority of the nonrespondents were people who refused to participate in the survey without giving any reason. Every nonresponding telephone number was replaced by another one with the same or similar characteristic until 1000 questionnaires were collected in each survey. The response rate was 32.8%, 34.2%, and 5.2%, respectively, in the 2007, 2012, and 2018 surveys. The 2018 survey response rate was significantly lower than the previous ones, but the representativeness of the survey was achieved. The factor that contributed most to such a low response rate was not answering the phone (19,200 people). The 2018 survey was conducted from December to January, which is a holiday period. There is also a growing reluctance to answer unwanted calls. Nowadays, hardly anyone without a publicly available telephone number responds to such calls [18].

All the subjects gave their informed consent before they participated in the study. The study was conducted in accordance with the Declaration of Helsinki, and the protocol was approved by the Ethics Committee of Wroclaw Medical University (ST 481/2010, ST C290.17.040).

### 2.2. Questionnaire

The study was a part of three national surveys prepared on the basis of our previous research concerning trends and patterns of health-related internet use [1,19,20,21,22,23,24,25]. The survey conducted in 2007, which was also part of a survey on WHO/European e-health consumer trends [24,26], contained 24 original questions in Polish compared to the surveys carried out in 2012 and 2017/18 that contained 22 questions each (see the questionnaires in the supplementary materials, Questionnaires S1 and S2). Due to the fact that there is only one official language commonly used in Poland, there was no other language version of the survey. This study was focused on one complex question, which was exactly repeated in each survey.

In the first part of the questionnaire, the respondents were asked if they would use their online EHR, assuming that they have access to the internet. The next part of this questionnaire concerned their willingness to pay approximately EUR 30 per year for such a service.

The respondent had the possibility to answer “yes” or “no”, with an additional option of “I do not know”.

Supplementary demographic data of respondents, such as sex, age, education, the size of the place of residence, or professional situation, were also obtained.

### 2.3. Data Analyses

A general group of all respondents and the three distinguished groups from the three surveys were taken into account for analyses. A descriptive analysis, followed by statistical analysis, was carried out in order to identify significant associations between the participant independent variables and changes in their attitude over the years, setting the trend. The statistical packages R software (version 3.6.3, 29 February 2020, R Foundation for Statistical Computing, Vienna, Austria) and G*Power software (version 3.1.9.6, G*Power—Erdfelder, Faul & Buchner, Düsseldorf, Germany) were used in the calculations. All tested variables were of a qualitative type.

For the analysis, the chi-square test of independence and homogeneity was used. Statistical hypotheses were verified at the significance level of 0.05, and, in each case, the test power (1−β) was calculated by the posthoc method. Then, 95% confidence intervals were determined for certain frequencies (attached in the supplementary materials, Table S1).

Subsequently, correspondence analysis was conducted. This method provides information similar to the interpretation of the results of factor analysis, but on qualitative variables. Analysis of statistics and figures proposed by this method allows simple and intuitive inference about the relationships occurring between the categories of variables. With the help of the correspondence analysis, the profile of the most likely EHR user was determined. A profile was defined for the respondents of the study in 2018 as it is important to determine the most current EHR user profile. 

Each of the variables used in the analysis of correspondence is a 2–5 categorical feature to ensure the best subsequent interpretation of the clusters on a two-dimensional figure. As a result of correspondence analysis carried out on a set of categorical variables, a two-dimensional graph is obtained, which is a set of points where each point corresponds to one category. The set of points can form clusters, i.e., subsets of points located closer to each other. The correspondence analysis method is rooted in the fact that the categories (points) belonging to clusters are interpreted as related to each other. 

Percentages at the coordinate axes indicate which dimension has a greater effect on the distance between the points. In our case, Dimension 1 is by far the most influential, so there are lines, L1 and L2, in the figure. These lines mark the boundaries of three hypothetical clusters around EHR category points: a, b, c.

## 3. Results

### 3.1. Characteristics of the Respondents

The study group consisted of 1598 women (53.3%) and 1402 men (46.7%). Three age groups were distinguished: young (15–35 years-old (y.o.), who constituted 1042 individuals (34.7%), middle-aged (36–59 y.o.), who accounted for 1257 people (41.9%), and older (60–94 y.o.), in the number of 701 respondents (23.4%). With regard to the size of the place of residence among the studied population, the distribution was comparable to big cities (31%), small towns (31%), and villages/rural areas (38%). This applied to both the entire population and the distribution in individual surveys carried out in 2007, 2012, and 2018. The vast majority of respondents (86%) lived with someone else.

Out of all respondents, 1912 people were interested in access to EHRs (63.7%), 996 individuals were not interested (33.2%), and 92 respondents had no opinion (3.1%). Additionally, 920 people agreed to pay for access to EHRs (30.7% of all respondents), and 48.1% of individuals from this group were interested in accessing EHRs. In contrast, 992 people did not agree to pay (33.1% of the studied group), and 51.9% of this group were interested in EHR. People who were not interested in accessing EHRs or had no opinion did not answer the question concerning consent to pay for access to EHRs.

Detailed information on the study population is provided in Table 1.

### 3.2. Interest in Access to EHRs

**Figure 1:**
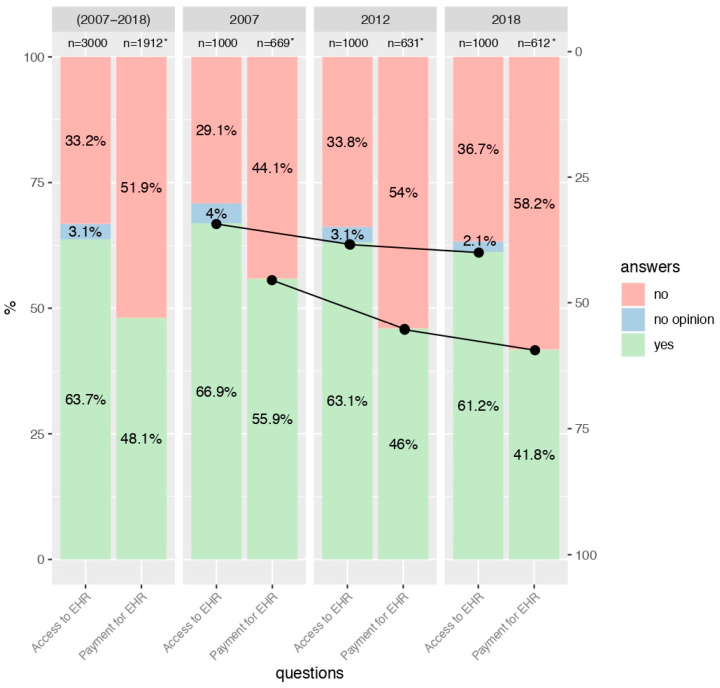
General interest in access to EHR and consent to payment for access to EHR in 2007, 2012 and 2018. * People who were not interested in accessing EHR or had no opinion, did not answer the question concerning consent to pay for access to EHR.

**Figure 2:**
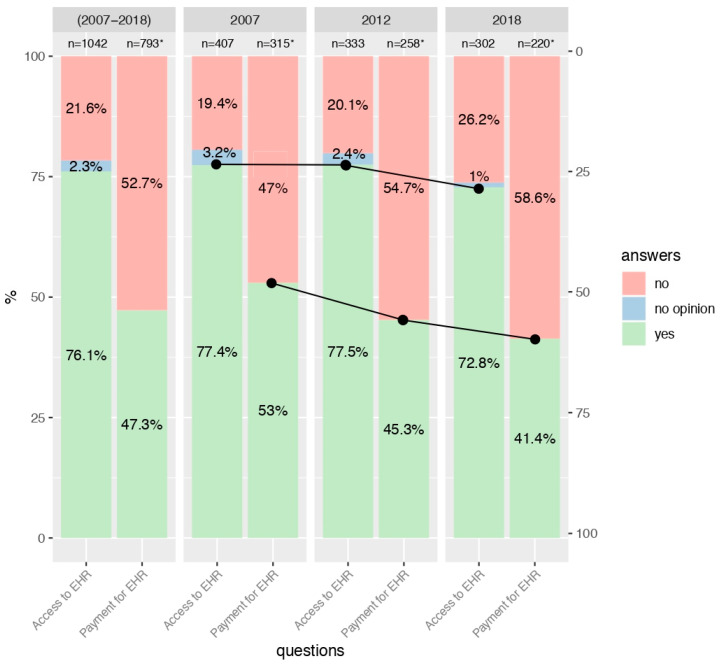
Interest in access to EHR and consent to payment for access to EHR among respondents aged 15–35 years in 2007, 2012 and 2018. * People who were not interested in accessing EHR or had no opinion, did not answer the question concerning consent to pay for access to EHR.

**Figure 3:**
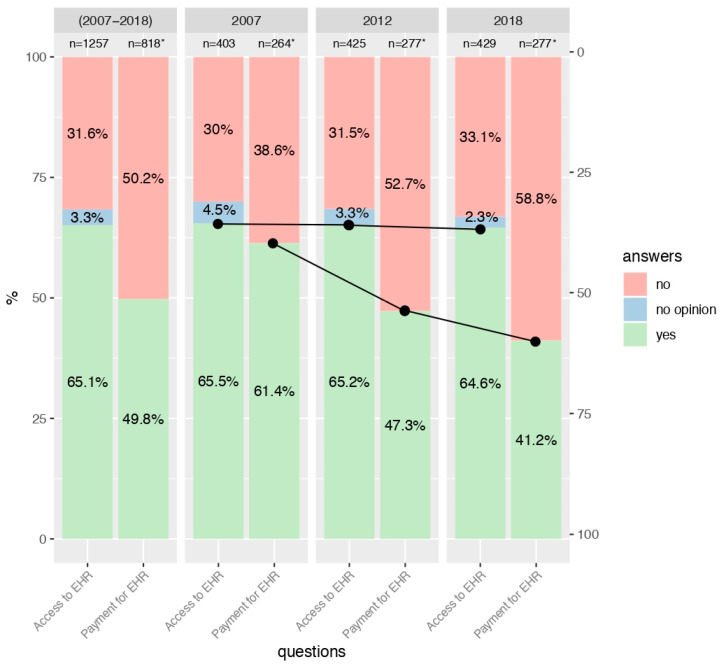
Interest in access to EHR and consent to payment for access to EHR among respondents aged 36–59 years in 2007, 2012 and 2018. * People who were not interested in accessing EHR or had no opinion, did not answer the question concerning consent to pay for access to EHR.

**Figure 4:**
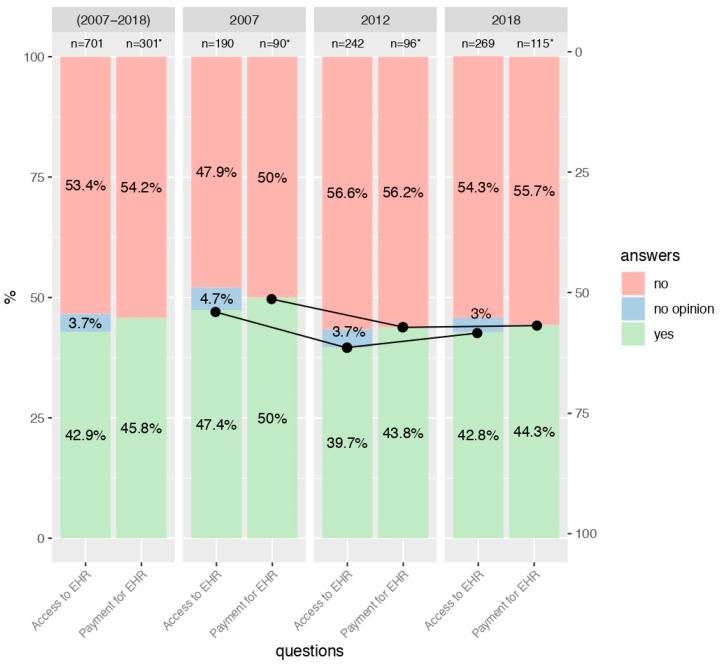
Interest in access to EHR and consent to payment for access to EHR among respondents aged 60+ years in 2007, 2012 and 2018. * People who were not interested in accessing EHR or had no opinion, did not answer the question concerning consent to pay for access to EHR.

Interest in access to EHRs significantly decreased in the general population in the years 2007, 2012, and 2018 (69.7%, 65.1%, and 62.5%, respectively; *p* = 0.003; see Figure 1). There was also a significant relationship between interest in access to EHRs and the age of the study participants (*p* < 0.001). Generally, the highest interest was shown by young individuals (76.1%, Figure 2), then by middle-aged respondents (65.1%, Figure 3), and the lowest level of interest was expressed by older people (42.9%, Figure 4).

Analyzing the individual age groups, interest in access to EHRs among young people in the two studies carried out in 2007 and 2012 remained relatively stable (77.4% and 77.5%). Nevertheless, a decrease to the level of 72.8% was detected in 2018 (see Figure 2). With regard to the group of middle-aged people, interest in access to EHRs remained relatively steady in subsequent years of study (65.5%, 65.2%, and 64.6%, respectively; see Figure 3), and in the group of older people, the observed trend fluctuated in a sinusoidal pattern (47.4%, 39.7%, and 42.8%; see Figure 4). For more details, see Table S3 in the supplementary materials.

### 3.3. Consent to Payment for Access to EHRs

Generally, consent to pay for access to EHRs was declared by 48.1% of the entire surveyed population. In the subsequent years, the level of acceptance of payment decreased significantly (55.9%, 46%, and 41.8%; *p* < 0.001). More information is presented in Figure 1. The average percentage of people in each age group declaring their consent to pay for access to EHRs was estimated at 47.3% for young people (Figure 2), 49.8% for middle-aged individuals (Figure 3), and 45.8% for older people (Figure 4). There was no statistically significant relationship between them (see Table S2 in the supplementary materials).

The trend in the number of people who would agree to pay for access to EHRs decreased in all age groups. In the group of young people, it declined from the level of 53.0% in 2007 to 45.3% in 2012 and to 41.4% in 2018 (*p* = 0.022; see Figure 2). With regard to the middle-aged respondents, it was 61.4%, 47.3%, and 41.2%, respectively (*p* < 0.001; see Figure 3). For the group of elderly individuals, it was the only age group where this distribution was not statistically significant. However, starting from 50% in the 2007 study, it collapsed to 43.8% in 2012 and remained almost constant at 44.3% in 2018 (see Figure 4). For more details, see Table S3 in the supplementary materials.

### 3.4. Impact of Sociodemographic Variables on Interest in Access to EHRs

Interest in access to EHRs was higher among men than women (66% versus 61.8%; *p* = 0.044), among those living with someone else than alone (64.1% versus 50.5%; *p* < 0.001), and when comparing people using a mobile phone to those who were not (64.6% versus 32.9%; *p* < 0.001). An increase in interest in access to EHRs was also associated with a higher level of education (*p* < 0.001), residency in urban areas (*p* < 0.001), and better subjective assessment of health (*p* < 0.001). It should be noted that the greatest interest was shown by people using the internet every day (76.4%; *p* < 0.001), followed by people using it at least once a year (63.8%; *p* < 0.001). It is interesting that as much as 43.4% of people who have never used the internet were still interested in accessing EHRs. Analyzing the group of people using the internet for health purposes (HI-users), the interest in accessing EHRs increased with a higher frequency of using the internet for health purposes (*p* < 0.001). In the group of general internet users, interest was demonstrated by 52% of people not using the internet for health purposes (non-HI-users). Access to EHRs gained the greatest popularity among students (78.7%) and the lowest interest among pensioners (45.7%); *p* < 0.001. Detailed data are included in Table 2.

### 3.5. Impact of Sociodemographic Variables on Consent to Payment for Access to EHRs

The only statistically significant factor affecting the consent to pay for access to EHRs was the frequency of using the internet for health purposes (*p* = 0.048). Most often, such consent was given by everyday HI-users. Similarly, as to interest in access to EHRs, it is surprising that 44.6% of non-HI-users were willing to pay for such access. The consent to pay for access to EHRs was not affected by sex, education, living with someone or alone, the size of the place of residence, professional situation, frequency of internet use, subjective assessment of health, or use of a mobile phone. All these variables had a significant impact on interest in access to EHRs, although they were not statistically significant in relation to consent to pay for such a service. More details are included in Table 3.

### 3.6. Profile of the Potential EHR User

**Figure 5:**
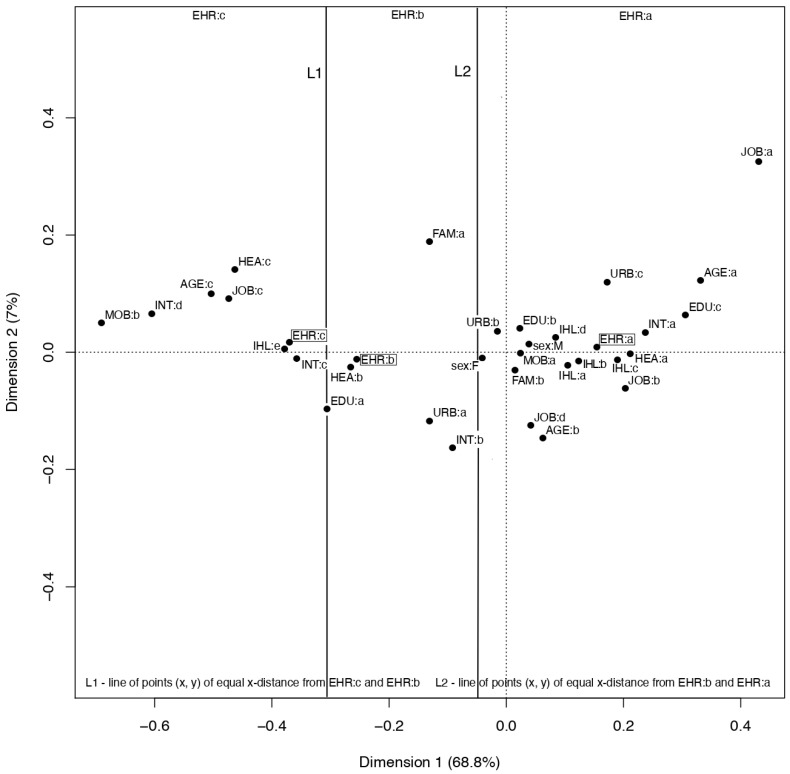
Profile of persons interested or not interested in access to EHR in 2018 year based on correspondence analysis (legend in Supplementary Materials, Table S4).

Correspondence analysis of interest in access to EHRs based on the most recent data from the 2018 survey presented three clusters of more frequent coexistence of categories of the studied variables: a (people interested in access to EHR), b (people not interested in access to EHR) and c (people with no opinion on access to EHR); see Figure 5.

Belonging to a young or middle-aged group, having secondary or higher education, living with a family in a small town or big city, studying, working, or perchance being unemployed, using a mobile phone, using the internet every day and any use of the internet for health purposes, and good or very good subjective health assessment were positively correlated with greater interest in access to EHRs.

On the other hand, living alone in the countryside, having primary education, using the internet at least once a month, and an average subjective assessment of health contributed to the reluctance to access EHR. 

Other characteristics, such as older age, being a pensioner, using the internet at least once a year or never and not using it for health purposes, not having a cell phone, and assessing health as bad, were typical of those who had no opinion about accessing EHRs.

The analysis of correspondence for the consent to pay for access to EHRs did not show any differences between the clusters for a given category (yes or no). In the figure, the two categories are close together, even sharing the same *x*-axis coordinate. This is the reason why it was impossible to identify the characteristics of the person who would most likely pay for access to EHRs (see Figure S1 in the supplementary materials).

## 4. Discussion

### 4.1. Trends Regarding Interest in Access to EHRs

As our study shows, a general interest in access to EHRs remains at a relatively high level. However, it has significantly decreased in studies carried out in 2007, 2012, and 2018. This fact seems to be awkward because the use of EHRs in healthcare is constantly increasing. On the other hand, the downward trend may be the result of several changes that have occurred in societies. It appears to be related, inter alia, to the commonly observed aging of the population, and the fact that older people use the internet less often [27,28]. Another important issue is that there is an increase in awareness among internet users, and a lot of data leaks have been publicized [29,30]. The fear of internet threats is growing [31]. Network security, despite its constant development, does not guarantee full data security [32,33]. It should also be considered that access to EHRs has been implemented in many countries around the world [11], so, as a result, a large proportion of patients have already experienced EHRs, which nowadays seems to have greater value for doctors than for patients [34,35].

Analyzing the individual age groups, it can be seen that the greatest interest in access to EHRs is shown by young people, but this has also decreased over the years. One of the reasons for this can be a growing pace of life [36,37]. Busy young people usually leave health decisions to their physicians without using EHRs personally, and they expect fast and effective service from a doctor [38,39]. Nevertheless, access to EHRs positively affects the satisfaction of patients who receive made-to-measure medical services. The next important factor explaining the lower interest among young people may be associated with the low morbidity in this age group [40]. In turn, the middle-aged group is the most stable in terms of trend analysis. According to many authors’ reports, middle-age is the time when the risk of developing lifestyle diseases increases significantly [41,42]. The need for multispecialist consultations in this group of patients has an impact on maintaining a high interest in access to EHRs [41,42]. Among older people, the sinusoidal trend may be due to the fact that many of them do not really understand what EHRs are exactly, but often want to keep up with news [43,44]. These people also have more free time that they can devote to an interest in health. Moreover, as also confirmed by other authors, the majority of them are afraid of social isolation and exclusion, which may explain the desire to keep up with the latest trends [44,45].

### 4.2. Trends Regarding Consent to Payment for Access to EHRs

Trends concerning consent to pay for access to EHRs are related to a decrease in general interest in this service. In subsequent studies carried out in 2008, 2012, and 2018, the overall acceptance of a fee for this service has gradually declined. 

Such a situation is observed in particular age groups as well. Among young people, a greater reluctance to make payments may be due to low earnings at the beginning of their careers and increasing spending when starting a new family [46,47]. According to many other authors, because of convenience and increasing demands for high comfort in life, they become independent later, so their health is cared for by their families [47,48]. Middle-aged people statistically showed the highest willingness to pay for access to EHRs, but at the same time, the highest downward trend year-on-year was observed in this group. The decrease can be associated with the popularization of employer-paid medical care subscriptions as an additional bonus to salary and the more frequent use of the private healthcare sector, where access to EHRs is often provided in the price of the service [49,50,51]. The percentage of older people agreeing to pay for access to EHRs has been relatively steady over the years. Health expenses are becoming the main cost of living in this age group and have the highest priority [52,53]. However, when comparing this age group with others, the biggest reluctance to pay for access to EHRs is visible. This may be related to their large expenditure on health [52,53].

In addition to the above, the study shows a very interesting phenomenon that even part of non-HI-users and those who do not use the internet at all are interested in accessing EHRs, and, furthermore, they even agree to pay for this service. Unfortunately, there is not enough data to explain this unclear finding. One of the explanations could be the fact that health is very valuable. Possibly, in their view, interest in accessing EHRs is simply health-oriented behavior and not a direct benefit for themselves [54,55,56]. Likewise, it may indicate their willingness to provide such access to family members or medical staff. It is also possible that after receiving such access, they would start using the internet for health and/or general purposes [54,55,56,57].

### 4.3. Impact of Sociodemographic Variables on Interest in Access and Consent to Pay for EHRs

The study shows that all analyzed sociodemographic variables had a statistically significant effect on interest in accessing EHRs. Men are more interested in access to EHRs, as confirmed by many studies. This is probably due to more frequent professional contact with e-services [58,59]. People living with someone else showed similar higher interest, which could be explained by the willingness to share information in the household and care for other family members [57,60]. The use of a mobile phone is also positively related to interest in access to EHRs as it is a sign of technological progress [61,62]. The positive relationships with higher education and living in a larger city may result from greater openness to new opportunities, being surrounded by all sorts of innovations, and greater awareness of healthy behaviors [63,64]. It also concerns students who travel a lot, therefore needing portable medical records [65,66]. Retirees are a group who is least interested in access to EHRs as they are the least computerized and most analog group in a digital world [67,68]. Subjective health assessment also influences the interest in access to EHRs, which increases with better assessments. People who assess their health poorly may be more resigned and indifferent, so they are usually not interested in access to EHRs [69,70,71].

On the basis of the correspondence analysis, it is possible to distinguish the characteristics of the person who would most likely have accessed EHRs recently. The so-called “super-user” of EHRs is a man or woman, aged 15–59, living with family in the city. He is well educated and is still studying or working. He believes that his health is at least good, he has a mobile phone, and he eagerly uses the internet for health purposes on a daily basis (see Figure 5).

In contrast to interest in accessing EHRs, the willingness to pay for such a service depends only on the use of the internet for health purposes. As many other authors have confirmed, frequent HI-users have better-defined needs and requirements, which results from better knowledge of the possibilities and availability of the e-health sector’s services [72,73]. Trying to obtain the most reliable information, they become a partner to the doctor in decisions regarding their health [74], which seems to positively affect their acceptance to pay for access to EHRs.

### 4.4. Comparison with Other Countries and Implication for the Future

Although there are not many similar recent studies from other countries, comparing the situation related to willingness to access EHRs is crucial. Referring to the publication by Paccoud, Baumann et al. from 2020, based on surveys conducted in Luxembourg, Belgium, France, and Germany, on average, 82.6% of respondents were interested in accessing EHRs compared to 63.7% in our study [75]. It is worth mentioning that although we do not have such data from Poland due to a lack of implementation of EHRs so far, in Luxembourg, Belgium, France, and Germany, only 7.5% of respondents actually have access to EHRs, and only 3.5% intend to use EHRs regularly [75]. In Western countries, as in Poland, the interest in access to EHRs is positively correlated with a higher education level, and negatively correlated with older age. In Western countries, women have expressed greater interest in access to EHRs, while in Poland, men were a bit more interested [75]. The general interest in access to EHRs is lower in Poland than in Western countries. However, it should be noted that physical access to EHRs in every country remains low.

Efforts from each side are undoubtedly needed to promote EHRs and implement them on a bigger scale. The government could create a coherent platform that is easy and intuitive to use. It could also financially support the computerization of healthcare entities. Medical institutions should create a common, unified network that is cloud-based, which must be secure [76]. It would also be a good idea to organize EHR training for personnel, demonstrating to them how to use EHRs easily and pointing to the capabilities and advantages [77]. On the other hand, patients should be informed about the benefits of using EHRs, such as ensuring continuity of care and its holistic nature, by enabling every physician who contributes to the diagnostic and therapeutic process to have full insight into the patient’s documentation [78]. This is also a convenience for patients, who will not have to provide, in person, the results of consultations from other specialists, which helps to avoid the increasingly frequent problem of polypharmacy [79].

### 4.5. Limitations

The study has several limitations. First of all, there were few variables, such as education, inhabitancy, and mobile phone use, that were not included in the survey in 2007. Therefore, it was not possible to fully observe how and if these characteristics changed over time and to analyze the corresponding designation of the trend line.

The method of sample collection, reporting, and analyzing data was exactly the same for all three surveys. However, the study did not follow the same individuals over the study period, and because of that, the observed changes could be analyzed only from the perspective of the general population or distinguished subgroups and cannot represent the attitudes and opinions of the individual participants in the study.

It should be noted that in the subsequent studies, there was an increased percentage of older people. Such a distribution, however, best reflects the changes taking place in the structure of an aging society and is necessary to maintain the representativeness of the study group.

The question about money and income is always a tricky one and is often associated with the feeling of discomfort. On the one hand, a large part of the population may not want to be considered poor or scanty, and on the other hand, another part of society may be trying to hide their assets. Therefore, it was not possible to relate the financial status of respondents with the consent to pay for access to EHRs.

Finally, it should also be mentioned that although the survey carried out in 2018 is recent, the current situation may already vary due to the rapid development of telemedicine in the course of the SARS–CoV-2 pandemic. As a result, there is a need for further studies that can precisely analyze the current situation.

## 5. Conclusions

The development of e-health services, which is progressing around the world, seems to be the answer to the needs of doctors and patients. New solutions are constantly being introduced. During our 11-year observation, our research has shown that both interest in access to EHRs and consent to pay for it has generally remained at the same level, with an average interest of over 60%, of which more than half of the respondents are willing to pay for this service. However, despite this, a slight decrease in the year-on-year trend line is noted. The youngest age group showed the highest general interest in access to EHRs, and middle-aged people were the most willing to pay for it. The group of older people accounted for the lowest percentage of affirmative answers in both categories. The sociodemographic factors that had the most distinct impact on interest in access to EHRs included higher education, being professionally active or being a student, having a high subjective health assessment, using a mobile phone, using the internet on a daily basis, and frequently using the internet for health purposes. The last factor was also the only one significantly influencing the greater willingness to pay for access to EHRs. In summary, there is still a big demand for EHRs, and it has been an invariable segment of the health market for many years. The majority of people are open to innovations in e-health services, especially when they care about their health. That is why it is so important to educate patients, introduce a unified system that is friendly for medical attendants, and firmly implement EHRs, which have great potential to improve healthcare significantly for both patients and doctors.

## Figures and Tables

**Table 1:** Characteristics of respondents in total and in subsequent years 2007, 2012, and 2018.

Characteristics of Respondents		Overall		Year of the Study					
		(n = 3000)		2007 (n = 1000)		2012 (n = 1000)		2018 (n = 1000)	
Characteristics	Categories	n	%	n	%	n	%	n	%
Age	(a) 15–35 years old	1042	34.7	407	40.7	333	33.3	302	30.2
	(b) 36–59 years old	1257	41.9	403	40.3	425	42.5	429	42.9
	(c) 60+ years old	701	23.4	190	19	242	24.2	269	26.9
Sex	(a) male	1402	46.7	484	48.4	476	47.6	442	44.2
	(b) female	1598	53.3	516	51.6	524	52.4	558	55.8
Education	(a) primary	693	34.6	0	NaN *	354	35.4	339	33.9
	(b) secondary	741	37.0	0	NaN *	368	36.8	373	37.3
	(c) higher	566	28.3	0	NaN *	278	27.8	288	28.8
Inhabitancy	(a) alone	281	14.1	0	NaN *	138	13.8	143	14.3
	(b) with someone else	1716	85.9	0	NaN *	860	86.2	856	85.7
Residence	(a) village/rural area	1136	38.0	372	37.2	377	38	387	38.7
	(b) small town (<100.000 residents)	926	30.9	299	29.9	300	30.2	327	32.7
	(c) big city (>100.000 residents)	931	31.1	329	32.9	316	31.8	286	28.6
Professional situation	(a) student	291	9.7	172	17.2	75	7.5	44	4.4
	(b) working	1662	55.5	504	50.5	564	56.6	594	59.4
	(c) pensioner	868	29.0	260	26	291	29.2	317	31.7
	(d) unemployed	175	5.8	63	6.3	67	6.7	45	4.5
Frequency of	(a) everyday	1503	50.2	414	41.5	507	50.9	582	58.2
internet usage	(b) at least once a month	634	21.2	216	21.6	222	22.3	196	19.6
	(c) at least once a year	69	2.3	37	3.7	15	1.5	17	1.7
	(d) never	788	26.3	331	33.2	252	25.3	205	20.5
Frequency of health internet usage	(a) everyday (b) at least once a month	74 1240	3.2 54.1	24 345	3.6 52.1	17 452	2.3 61.1	33 443	3.7 49.8
	(c) at least once a month	576	25.1	147	22.2	184	24.9	245	27.5
	(d) less than once a year	48	2.1	0	0	0	0	48	5.4
	(e) never	354	15.4	146	22.1	87	11.8	121	13.6
Subjective health	(a) good/very good	1737	58.2	592	59.4	575	57.8	570	57.5
assessment	(b) average	1036	34.7	338	33.9	351	35.3	347	35
	(c) bad/very bad	209	7.0	66	6.6	69	6.9	74	7.5
Interest in access to EHRs	(a) yes	1912	63.7	669	66.9	631	63.1	612	61.2
	(b) no	996	33.2	291	29.1	338	33.8	367	36.7
	(c) I do not know	92	3.1	40	4	31	3.1	21	2.1
Consent to payment for access to EHRs **	(a) yes	920	48.1	374	55.9	290	46	256	41.8
	(b) no	992	51.9	295	44.1	341	54	356	58.2
Using a cell phone	(a) yes	1848	92.4	0	NaN *	891	89.1	957	95.7
	(b) no	152	7.6	0	NaN *	109	10.9	43	4.3

* NaN—the value could not be calculated due to lack of data in the 2007 study. ** People who were not interested in accessing electronic health records (EHRs) or had no opinion did not answer the question concerning consent to payment for access to EHRs.

**Table 2:** Impact of sociodemographic variables on the distribution of answers about interest in EHRs.

Characteristics of Respondents		Interest in Access to EHRs							
		(a) yes		(b) no		(c) I don’t know		*p*	
Variable	Categories	n	%	n	%	n	%	1 − β	
Sex	(a) male	925	66	440	31.4	37	2.6	0.044	
	(b) female	987	61.8	556	34.8	55	3.4	0.604	
Education	(a) primary	334	48.2	334	48.2	25	3.6	0	*
	(b) secondary	481	64.9	239	32.3	21	2.8	1	
	(c) higher	428	75.6	132	23.3	6	1.1		
Inhabitancy	(a) alone	142	50.5	125	44.5	14	5	0	*
	(b) with someone else	1100	64.1	578	33.7	38	2.2	0.992	
Residence	(a) village/rural area	650	57.2	447	39.3	39	3.4	0	*
	(b) small town (<100.000 residents)	612	66.1	290	31.3	24	2.6	1	
	(c) big city (>100.000 residents)	648	69.6	254	27.3	29	3.1		
Professional situation	(a) student	229	78.7	57	19.6	5	1.7	0	*
	(b) working	1175	70.7	440	26.5	47	2.8	1	
	(c) pensioner	397	45.7	438	50.5	33	3.8		
	(d) unemployed	109	62.3	59	33.7	7	4		
Frequency of internet usage	(a) everyday	1148	76.4	326	21.7	29	1.9	0	*
	(b) at least once a month	376	59.3	236	37.2	22	3.5	1	
	(c) at least once a year	44	63.8	25	36.2	0	0		
	(d) never	342	43.4	405	51.4	41	5.2		
Frequency of	(a) everyday	58	78.4	14	18.9	2	2.7	0	*
health internet usage	(b) at least once a month	918	74	296	23.9	26	2.1	1	
	(c) at least once a year	411	71.4	155	26.9	10	1.7		
	(d) less than one a year	30	62.5	17	35.4	1	2.1		
	(e) never	184	52	155	43.8	15	4.2		
Subjective health assessment	(a) good/very good	1221	70.3	465	26.8	51	2.9	0	*
	(b) average	585	56.5	421	40.6	30	2.9	1	
	(c) bad/very bad	97	46.4	101	48.3	11	5.3		
Using a cell phone	(a) yes	1193	64.6	613	33.2	42	2.3	0	*
	(b) no	50	32.9	92	60.5	10	6.6	1	

* *p* = 0 means *p* < 0.001.

**Table 3:** Impact of sociodemographic variables on the distribution of answers about consent to payment for access to EHRs.

Characteristics of Respondents		Consent to Payment for Access to EHRs *				
		(a) yes		(b) no		*p*
Variable	Categories	n	%	n	%	1 − β
Sex	(a) male	450	48.6	475	51.4	0.686
	(b) female	470	47.6	517	52.4	0.069
Education	(a) primary	148	44.3	186	55.7	0.985
	(b) secondary	211	43.9	270	56.1	0.053
	(c) higher	187	43.7	241	56.3	
Inhabitancy	(a) alone	55	38.7	87	61.3	0.213
	(b) with someone else	491	44.6	609	55.4	0.238
Residence	(a) village/rural area	330	50.8	320	49.2	0.15
	(b) small town (<100.000 residents)	294	48	318	52	0.396
	(c) big city (>100.000 residents)	294	45.4	354	54.6	
Professional situation	(a) student	100	43.7	129	56.3	0.528
	(b) working	575	48.9	600	51.1	0.210
	(c) pensioner	191	48.1	206	51.9	
	(d) unemployed	54	49.5	55	50.5	
Frequency of internet usage	(a) everyday	537	46.8	611	53.2	0.521
	(b) at least once a month	191	50.8	185	49.2	0.213
	(c) at least once a year	21	47.7	23	52.3	
	(d) never	170	49.7	172	50.3	
Frequency of	(a) everyday	30	51.7	28	48.3	0.048
health internet usage	(b) at least once a month	461	50.2	457	49.8	0.694
	(c) at least once a year	174	42.3	237	57.7	
	(d) less than one a year	11	36.7	19	63.3	
	(e) never	82	44.6	102	55.4	
Subjective health assessment	(a) good/very good	576	47.2	645	52.8	0.548
	(b) average	292	49.9	293	50.1	0.151
	(c) bad/very bad	46	47.4	51	52.6	
Using a cell phone	(a) yes	529	44.3	664	55.7	0.194
	(b) no	17	34	33	66	0.196

* People who were not interested in accessing EHRs or had no opinion did not answer the question concerning consent to pay for access to EHRs.

## Supplementary Materials

The following are available online at https://www.mdpi.com/1660-4601/17/17/6165/s1. Table S1: Distribution of “yes” and “no” answers about interest in access to EHRs in subsequent trials in total and separately for each age group, with 95% confidence intervals (CI1, CI2). Table S2: Distribution of answers about interest in access to EHRs and consent to payment for such access in age groups for all trials combined. Table S3: Distribution of answers about interest in access to EHRs and consent to payment for such access in subsequent trials, separately for each age group. Table S4: Legend to Figure 5 and Figure S1. Figure S1: Profile of persons willing or not willing to pay for access to EHRs in 2018 based on correspondence analysis (legend in supplementary files, Table S4). Questionnaire S1: 2007 year survey questionnaire (originally in English). Questionnaire S2: 2012 and 2018 survey questionnaires (translated to English).
