# Supplementary material for: Access to an Electronic Health Record: A Polish National Survey
Source: Int J Environ Res Public Health. 2020 Aug 25;17(17):6165. doi: 10.3390/ijerph17176165 (PMC7503705; doi:10.3390/ijerph17176165)
Supplement: Supplementary file 1 [file ijerph-17-06165-s001.pdf]

International Journal of
Environmental Research and Public Health

MDPI

Article

Access to an Electronic Health Record: A Polish National Survey

Maria Magdalena Bujnowska-Fedak * and Łukasz Wysoczański

[1] Department of Family Medicine, Wroclaw Medical University, 51-141 Wrocław, Poland; lukasz.wysoczanski@student.umed.wroc.pl
* Correspondence: maria.bujnowska-fedak@umed.wroc.pl; Tel.: +48-71-3266876 (PL)

Received: 14 July 2020; Accepted: 18 August 2020; Published: date—

Supplementary Materials

Table S1. Distribution of "yes" and "no" answers about interest in access to EHR in subsequent trials in total and separately for each age group with 95% confidence intervals (CI1, CI2).

			Study year												
	Age		2007				2012				2018				*p*
Variable	group	Categories	n	%	CI1	CI2	n	%	CI1	CI2	n	%	CI1	CI2	1-β
Interest in access to EHR	all	(a) yes	669	69.7	66.7	72.6	631	65.1	62.0	68.1	612	62.5	59.4	65.6	0.003
		(b) no	291	30.3	27.4	33.3	338	34.9	31.9	38.0	367	37.5	34.4	40.6	0.864
	15—35 y.o.	(a) yes	315	79.9	75.6	83.8	258	79.4	74.6	83.7	220	73.6	68.2	78.5	0.099
		(b) no	79	20.1	16.2	24.4	67	20.6	16.3	25.4	79	26.4	21.5	31.8	0.471
	36—59 y.o.	(a) yes	264	68.6	63.7	73.2	277	67.4	62.6	71.9	277	66.1	61.4	70.6	0.758
		(b) no	121	31.4	26.8	36.3	134	32.6	28.1	37.4	142	33.9	29.4	38.6	0.094
	60+ y.o.	(a) yes	90	49.7	42.2	57.2	96	41.2	34.8	47.8	115	44.1	37.9	50.3	0.218
		(b) no	91	50.3	42.8	57.8	137	58.8	52.2	65.2	146	55.9	49.7	62.1	0.326

Table S2. Distribution of answers about interest in access to EHR and consent to payment for such an access in age groups for all trials combined.

		Age group							
		15—35 y.o.		36—59 y.o.		60+ y.o.		*p*	
Variable	Categories	n	%	n	%	n	%	1-β	
Interest in access to EHR	(a) yes	793	76.1	818	65.1	301	42.9	0	*
	(b) no	225	21.6	397	31.6	374	53.4	1	
	(c) I don't know	24	2.3	42	3.3	26	3.7		
Consent to payment for access to EHR	(a) yes	375	47.3	407	49.8	138	45.8	0.423	
	(b) no	418	52.7	411	50.2	163	54.2	0.199	

* $p = 0$ means $p < 0.001$.

Table S3. Distribution of answers about interest in access to EHR and consent to payment for such an access in subsequent trials separately for each age group.

			Study year						
	Age		2007		2012		2018		*p*
Variable	group	Categories	n	%	n	%	n	%	1-β
Interest in access to EHR	15—35 y.o.	(a) yes	315	77.4	258	77.5	220	72.8	0.078
		(b) no	79	19.4	67	20.1	79	26.2	0.629
		(c) I don't know	13	3.2	8	2.4	3	1.0	
	36—59 y.o.	(a) yes	264	65.5	277	65.2	277	64.6	0.479
		(b) no	121	30.0	134	31.5	142	33.1	0.236
		(c) I don't know	18	4.5	14	3.3	10	2.3	
	60+ y.o.	(a) yes	90	47.4	96	39.7	115	42.8	0.407
		(b) no	91	47.9	137	56.6	146	54.3	0.319
		(c) I don't know	9	4.7	9	3.7	8	3.0	
Consent to payment for access to EHR	15—35 y.o.	(a) yes	167	53.0	117	45.3	91	41.4	0.022
		(b) no	148	47.0	141	54.7	129	58.6	0.696
	36—59 y.o.	(a) yes	162	61.4	131	47.3	114	41.2	0 *
		(b) no	102	38.6	146	52.7	163	58.8	0.994
	60+ y.o.	(a) yes	45	50.0	42	43.8	51	44.3	0.638
		(b) no	45	50.0	54	56.2	64	55.7	0.124

* $p = 0$ means $p < 0.001$.

Table S4. Legend to the Figure 5 and the Figure S1.

			EHR clusters		
**Variable**	**Code**	Description	a	b	c
Age (years)	AGE:a	15—35	●		
	AGE:b	36—59	●		
	AGE:c	60+			●
Sex	sex:F	female	●		
	sex:M	male	●		
Education	EDU:a	primary		●	
	EDU:b	secondary	●		
	EDU:c	higher	●		
Inhabitancy	FAM:a	alone		●	
	FAM:b	with family	●		
Residence	URB:a	village/rural area		●	
	URB:b	small town	●		
	URB:c	big city	●		
Professional situation	JOB:a	student	●		
	JOB:b	working	●		
	JOB:c	pensioner			●
	JOB:d	unemployed	●		
Frequency of the Internet usage	INT:a	everyday	●		
	INT:b	at least once a month		●	
	INT:c	at least once a year			●
	INT:d	never			●
Frequency of Health Internet usage	IHL:a	everyday	●		
	IHL:b	at least once a month	●		
	IHL:c	at least once a year	●		
	IHL:d	less than once a year	●		
	IHL:e	never			●
Subjective health assessment	HEA:a	bood/very good	●		
	HEA:a	average		●	
	HEA:a	bad/very bad			●
Interest in access to EHR	EHR:a	yes	●		
	EHR:b	no		●	
	EHR:c	I don't know			●

Consent to payment for access to EHR	EHP:a	yes	- *	- *	- *
	EHP:b	no	- *	- *	- *
Using a cell phone	MOB:a	yes	●		
	MPB:b	no			●

* No clusters.

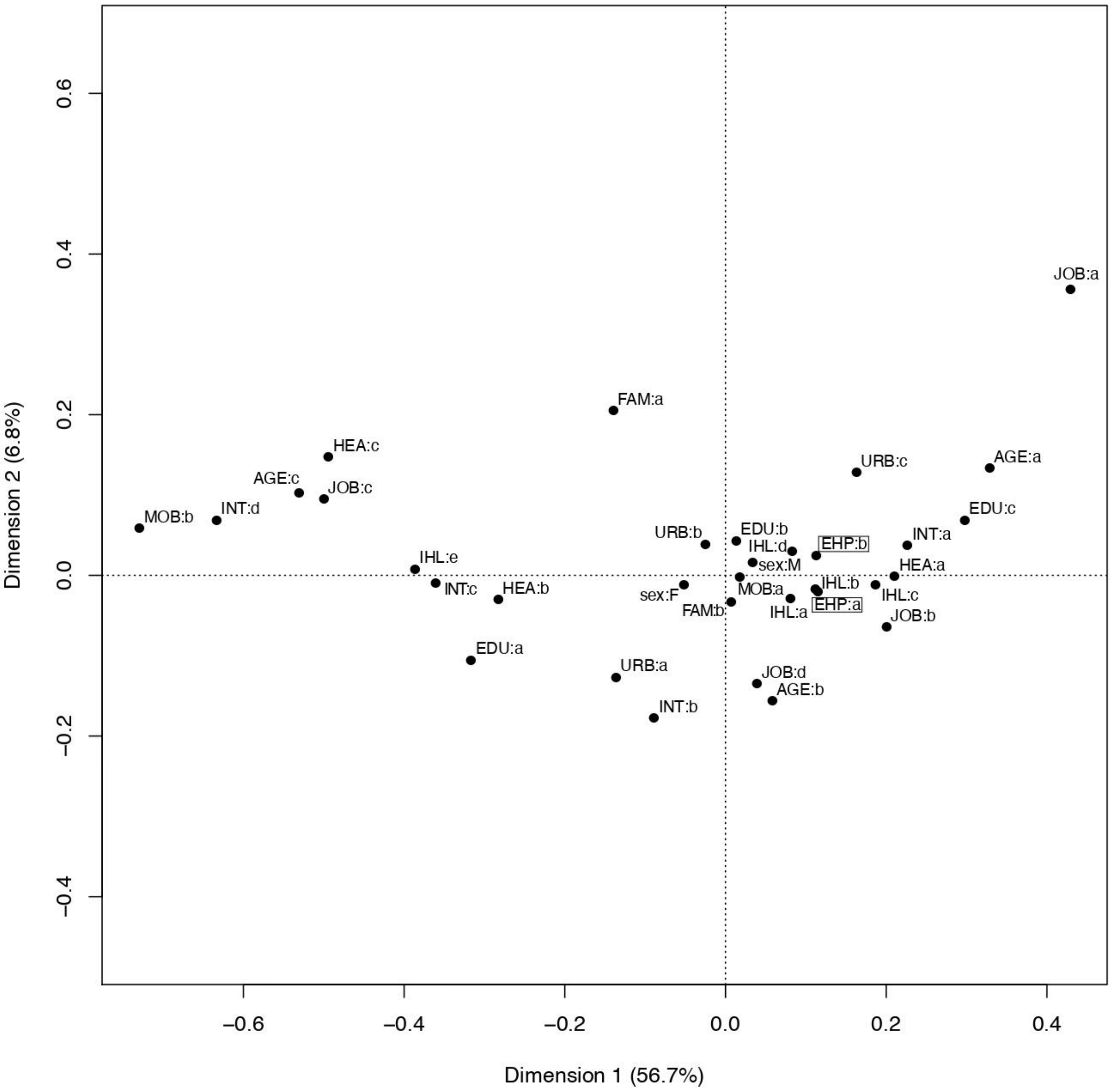

Figure S1. Profile of persons willing or not willing to pay for access to EHR in 2018 year based on correspondence analysis (legend in Supplementary Materials, Table S4).

Questionnaire S1. 2007 year survey questionnaire (originally in English). Attached as PDF – "Questionnaire S1.pdf".

Questionnaire S2. 2012 and 2018 years survey questionnaire (translated to English). Attached as PDF – "Questionnaire S2.pdf".

© 2020 by the authors. Submitted for possible open access publication under the terms and conditions of the Creative Commons Attribution (CC BY) license (http://creativecommons.org/licenses/by/4.0/).
